# The mobility limitation in healthy older people is due to weakness and not slower muscle contractile properties

**DOI:** 10.1371/journal.pone.0253531

**Published:** 2021-06-18

**Authors:** Hans Degens, Julia Attias, Daniel Evans, Frederick Wilkins, Emma Hodson-Tole

**Affiliations:** 1 Faculty of Science and Engineering, Research Centre for Musculoskeletal Science & Sports Medicine, Manchester Metropolitan University, Manchester, United Kingdom; 2 Institute of Sport Science and Innovations, Lithuanian Sports University, Kaunas, Lithuania; Universidade Federal de Mato Grosso do Sul, BRAZIL

## Abstract

The maximal power generating capacity of a muscle declines with age and has a negative impact on the performance of daily life activities. As muscle power is the product of force and velocity, we recruited 20 young (10 men, 10 women: 20–31 years) and 20 older (10 men, 10 women: 65–86 years) people to investigate which of these components contributes to the lower power and performance in old age. After determination of the maximal isometric knee extension torque (MVC), they performed a countermovement jump (CMJ) in 1) the normal situation (normal), 2) with an extra load of 15% body weight (loaded) and 3) 15% lower body weight (unloaded with a pulley system), and a timed up-and-go test (TUG) in the normal or loaded condition. The TUG and CMJ performance was lower in old than young participants (p<0.001). Below a critical CMJ peak power of ~23.7 W·kg^-1^ TUG showed a progressive decrease. The CMJ take-off velocity (V_off_) in the normal condition was lower in old than young participants (p<0.001). However, the V_off_ *vs*. body weight/MVC relationship of the normal, loaded and unloaded data combined was similar in the old and young participants and fitted the Hill equation (R^2^ = 0.396). This indicates that 1) only when peak power drops below a critical threshold TUG becomes impaired and 2) there was no evidence for intrinsic slowing of the muscle contractile properties in older people, but rather the older people were working on a slower part of the force-velocity relationship due to weaker muscles.

## Introduction

Even healthy people older than 65 years show some decrement in the performance of the timed up-and-go (TUG) and 6-minute walk test (6MWT) [1,2]. Although they may not have problems with daily life activities, it has been shown that a lower score of mobility tests in healthy older people was predictive for the development of future mobility limitations [3]. It is therefore important to understand the underlying factors that lead to a poorer performance in the TUG and other tests for the ability to perform daily life activities.

Mobility involves skeletal muscle activity and the significance of muscle is reflected by the association of self-reported physical disability with a low muscle mass [4]. It should be noted, however, that muscle strength shows a proportionally larger decline than muscle mass during ageing, as reflected by a lower specific tension in old than young individuals [5,6] and in healthy older people there was no relationship between muscle mass and performance of the TUG and 6MWT [1]. There may even be an age-related decrease in power generating capacity without a concomitant significant decrease in muscle size [7]. Given that muscle strength [8] and power are more important determinants than muscle mass for mobility limitations [9] and the maximal rate of stair ascent [10] in the elderly, it is important to understand the factors that contribute to lower peak power in old age.

The maximal power generating capacity of a muscle is determined by the maximal isometric force, the maximal shortening velocity and the curvature of the force-velocity relationship. Although the age-related loss of muscle strength contributes to the loss of power generating capacity, it has been reported that the maximum angular velocity of knee extension was a better predictor of physical function than isometric strength [11]. Part of the loss of peak power in old age may thus be attributable to a slowing of the contractile properties.

**Fig 1:**
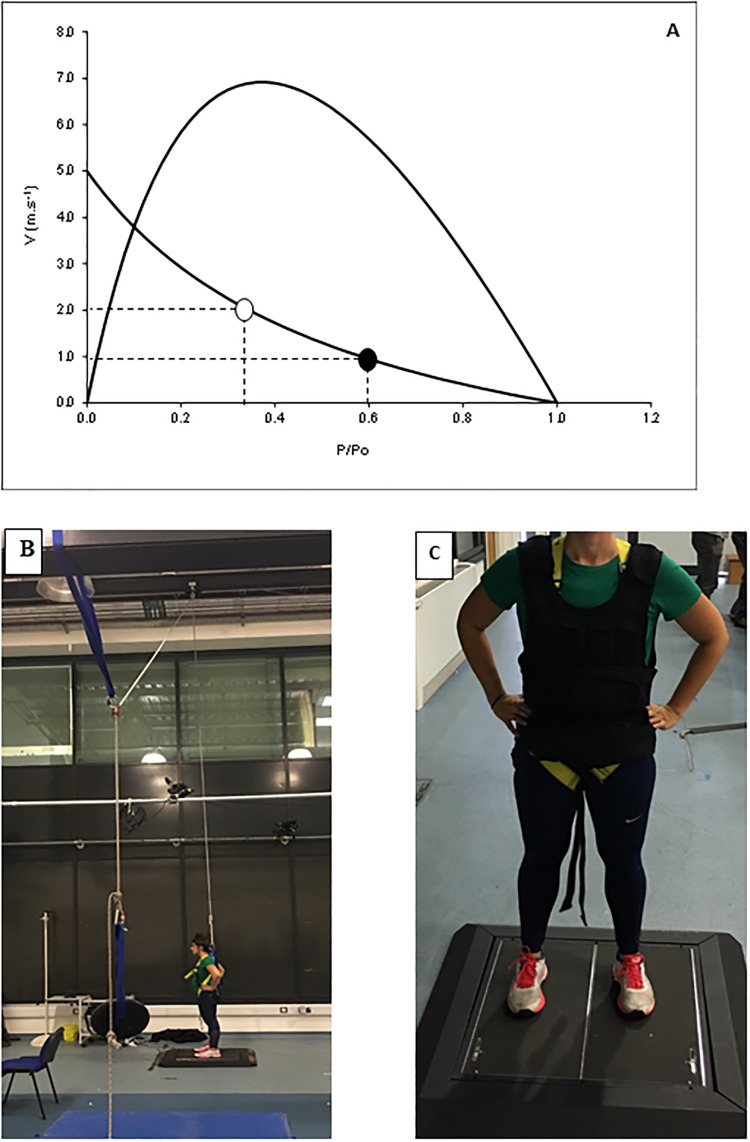
A) Force-velocity relationship illustrating that a higher body weight as a proportion of the maximal isometric force (P/Po) in an older (open symbol) than a young-adult person (closed symbol) results in a slower take-off velocity during a countermovement jump, even when the muscle contractile properties are unaltered. Participant set up for the B) unloaded condition, where a pulley system with counterweights was assembled. The other end of the rope was attached to the top of the harness of the participant enabling a 15% body weight (BW)-assisted jump and C) loaded condition, where 0.5-kg sandbags were added to a vest until 15% additional BW was acquired.

An age-related reduction in shortening velocity may well result from the preferential atrophy of faster type II fibres during ageing [12,13] and further contribute to the lower power generating capacity of old muscle as type II fibres have a less curved force-velocity relationship than type I fibres [14]. In line with these changes often seen during ageing, is the observation that the velocity at which optimal power is produced during leg press is lower in old age [15]. In addition, it has been suggested that the higher take-off velocity (V_off_) at a given body weight: maximal knee extensor torque ratio (BW:MVC) in young than old indicated that the lower peak power during a countermovement jump was due to slower contractile properties of the older muscle [1]. While this suggests that the contractile properties of the muscle are slower in old age, it is at least in theory possible that the lower V_off_ is just a consequence of the muscles of the older people working at a slower part of the force-velocity relationship [16], rather than an age-related slowing of the muscle contractile properties *per se* (illustrated in Fig 1A).

The aim of the present study is to assess whether part of the lower power generating capacity in old age is indeed attributable to slower contractile properties. To investigate this, we unloaded the muscles of older people and loaded the muscles of young people so both jump at a similar body weight to force ratio. We hypothesise that older people will still have a lower V_off_ than young people when the BW:MVC ratio is similar to that seen in young people. We hypothesise that a) muscle power generating capacity is lower in the older *vs*. the younger participants; b) that functional performance, as determined with the timed up-and-go (TUG test), is positively correlated to peak muscle power and c) that the decrease in peak power and thus performance is attributable to both slower contractile properties and loss of strength. We choose the TUG test as an indicator of functional performance as this test is more dependent on muscle power than e.g. the 6MWT that is primarily determined by percentage maximal heart rate [2].

## Methods

### Participants

Healthy young participants (10 men, 10 women) were recruited from among the university student population and healthy older participants (10 men, 10 women) were recruited from the local community. Participant characteristics are presented in Table 1. The study was approved by the local ethics committee of the Manchester Metropolitan University (ref: 9502) and abided to the Declaration of Helsinki. Written informed consent was obtained from each participant before participating in the study. The individual pictured in Fig 1 has given written informed consent (as outlined in PLOS consent form) to publish her picture. Exclusion criteria included pregnancy and/or breastfeeding, known neurological, cardiovascular or respiratory disease, or musculoskeletal injuries in the last 6 months. All participants were recreationally active.

**Table 1:** Participant characteristics.

	YM	OM	YW	OW
**Age**	28±3.6	72±5.9^Y^	27±4.5	69±3.4^Y^
**Height (cm)**	178±9	180±4	166±10^M^	160±6^M^
**BW (kg)**	82.7±10.8	84±8.7	61.5±5^M^	61.4±5.7^M^
**BMI (kg·m**^**-2**^**)**	25.9±2.8	25.9±3.3	22.5±1.9^M^	24.2±3.1^M^
**Body Fat (%)**	17±5.1	22.8±5.5^Y^	26.4±3.7^M^	32.2±5.7^M^^,^^Y^
**MVC (N·m)**	217±45	119±40^Y^	147±55^M^	71±24^M^^,^^Y^
**Peak Power·BW**^**-1**^ **(W·kg**^**-1**^**)**	49.3±8.6	29.2±5.4^Y^	38.1±6.5^M^	26.5±4.7^Y^^,^^M^

YM: Young men; OM: Older men; YW: Young women; OW: Older women; BW: Body weight; BMI: Body mass index; MVC: Maximal voluntary isometric knee extension torque.

^M^: Different from men at p≤0.008;

^Y^: Different from young at *p*≤0.001. Each group *n* = 10; data are mean±SD.

### Anthropometrics

Body weight (BW) was recorded on a digital scale (Seca, Switzerland) while the participants wore loose fitting gym clothing. Height was measured with a portable stadiometer to the nearest 0.1 cm (Seca, Switzerland). The body mass index (BMI) was calculated by dividing body weight in kilograms by the square of their height in meters. Body fat percentage was recorded using a handheld bioelectrical impedance monitor (model BF306, Omron healthcare LTD, Japan).

### Isometric maximal voluntary contraction torque

Knee extension torque of the right leg was measured as described previously [17]. Participants were seated on a custom-build rigid chair with the knee and hip joints at 90° flexion (full extension = 0°), with the hips strapped to minimize extraneous movements. Before determination of the maximal voluntary contraction (MVC) torque, the participants performed a warm-up consisting of 3-s intermittent incremental submaximal isometric contractions. During the subsequent determination of MVC, participants received visual feedback of the torque developed and strong verbal encouragement to produce maximal efforts lasting 3 s. The participants performed three MVCs with 60 s rest between contractions. The lever length was measured from the condyle of the knee to the attachment of the force transducer and the strongest of the three contractions was used to calculate the MVC torque.

### Peak muscle power

Maximal-effort countermovement vertical jumps (CMJ) were performed on a force platform (Leonardo Mechanograph GRFP STD, Novotec Medical, Pforzheim, Germany) to assess peak leg extension muscle power, using Newtonian physics [18]. The sampling rate of the force signal was 500 Hz and the resolution 0.01 N. Prior to performing any CMJs, participants performed a warm-up consisting of 10 squats, 10 lunges and 10 jumping jacks. Then they performed three CMJs with a 60-s rest interval between jumps. The CMJs were performed under three conditions: normal body weight (BW) (normal); 15% additional BW (loaded) and with 15% BW-support (unloaded) in a random order. After the set-up of each condition, participants were shown how to perform the CMJ by the experimenter and performed 2–3 practice jumps in each condition. During the practice jumps they were instructed to not attempt maximal effort, but to go through the motions and practice getting themselves off the ground and land on two feet in the same position as the take-off position and immediately after hitting the ground, bend the knees to minimise the risk of injury. Participants had 2–3 min rest between conditions [19].

The normal condition comprised wearing gym clothes plus a harness (which was worn during all three conditions over the clothes for standardisation). The total weight of the participant in this condition was classified as “normal”. For the loaded condition, a weighted vest equivalent to 15% of the BW was provided (Fig 1a). Weights of 0.5 kg (in the form of sandbags) were added as necessary. For the unloaded condition, a pulley system was utilised to create a counterweight system. One end of the rope was attached to the participant’s harness and the other end had weights equivalent to 15% BW (Fig 1b). In each condition, the CMJ with the greatest peak power was used for analysis. The peak power of the concentric phase, both in absolute terms (Watts) and as a function of body weight (W·kg^-1^), and peak ground reaction force (kN) were given by the software of the force platform. Jump velocity at take-off (V_off_ in m.s^−1^) were determined by the following calculation:

$$V_{\text{off}}\;=\;a\frac{t_{\text{f}}}{2}$$ (1)

Where ‘*a*’ is the gravitational acceleration (9.81 m·s^−2^) and ‘*t*_f_’ the flight time of the jump. The flight time was the time from take-off (no force recorded on the platform) until landing.

### Timed up-and-go

The timed up-and-go (TUG) was performed as described previously [1] in the normal and loaded condition. Participants were instructed to get up from a standard chair (44 cm high, no arm rests) and walk as quickly as possible, without running, around a cone 3 m away and return to the sitting position on the chair. Each participant performed the task three times per condition, with the fastest of the three trials in each condition being recorded. Participants rested for 30 s between trials.

### Statistical analysis

Data were analysed using SPSS statistics V24 (IBM, Armonk, NY). If a Shapiro-Wilk test showed that the data were not normally distributed they were log-transformed. A two-way univariate ANOVA with as between factors age and sex was used to compare differences in anthropometry, body fat percentage and MVC. Repeated-measures ANOVA was used to examine the effects of the within- (load) and between-participant (age and sex) effects. A significant age × load and sex × load interaction indicated that the effect of load differed between old and young participants, or men and women, respectively. Three-way interactions were excluded. To correct for multiple pairwise comparisons, a Bonferroni correction was applied. Based on previously published data on power and MVC [1] we calculated that a sample size of 6 people was sufficient to detect with two-sided tests an expected age-related difference 50% with a β of 80% and an α of 0.05. Ten people per group is therefore expected to provide sufficient statistical power for the current study.

A mixed effects linear model was used to determine the relationship between TUG and peak power per BW_adj_ in both young and old age groups, with subject included as the random factor. The model was constructed in R ×64 4.0.3 [20], using the *lme* function in the *nlme* package [21]. This enabled calculation of the association, while also accounting for the lack of independence between data points given the repeated measures nature of the data set. A first-order autoregressive structure (*corAR1*) was therefore included in the model. The same approach was used to determine the association between TUG and MVC per BW_adj_ and V_off_, as well as between V_off_ and MVC per BW_adj_.

Data were expressed as mean±SD. Significant differences were considered as *p*<0.05.

## Results

### Participant characteristics

Participant characteristics are shown in Table 1. Men were taller, had a higher body weight and BMI (*p*≤0.008) and a lower body fat percentage (*p*<0.001) than women. The body fat percentage was higher in older than younger men and women (*p* = 0.001).

In Table 1 it can also be seen that the MVC was higher in men than women (*p*<0.001) and lower in the old than young participants (*p*<0.001). The peak power per body weight was lower in old than young men and women (*p*<0.001) and was higher in men than women, irrespective of age (*p* = 0.004; Table 1).

### Countermovement jump and timed up-and-go

While men demonstrated higher peak power than women, and the power was lower in old than young participants (*p*<0.001). There was no significant effect of load on the peak power generated during a CMJ in men, but in women the power was lower in the unloaded than the normal condition (*p* = 0.005; Table 2).

**Table 2:** Countermovement jump parameters at UNLOADED, NORMAL and LOADED conditions in YM: Young men; OM: Older men; YW: Young women; OW: Older women.

	YM	OM	YW	OW
	UNLOADED			
**Peak Power (W)**	4232±589	2565±810^Y^	2293±667^M^	1326±327^M^^,^^Y^
**GRF (kN)**	1.79±0.26	1.50±0.14^Y^	1.19±0.13^M^	1.04±0.16^M^
**V**_**off**_ **(m·s**^**-1**^**)**	3.27±0.25	2.49±0.31^Y^	2.70±0.32^M^	1.82±0.23^M^^,^^Y^
**Jump height (m)**	0.49±0.18	0.28±0.09^Y^	0.36±0.08^M^	0.15±0.05^M^^,^^Y^
**TUG (s)**	/	/	/	/
	NORMAL			
**Peak Power (W)**	4085±670	2493±569 ^Y^	2390±463^1^^,^^M^	1643±323^1^^,^^M^^,^^Y^
**GRF (kN)**	2.02±0.30^1^	1.59±0.23^1^^,^^Y^	1.34±0.21^1^^,^ ^M^	1.19±0.18^1^^,^ ^M^
**V**_**off**_ **(m·s**^**-1**^**)**	2.65±0.22	1.80±0.35^1^^,^^Y^	2.19±0.21^M^	1.38±0.26^1^^,^^M^^,^^Y^
**Jump height (m)**	0.36±0.07	0.19±0.07^1^^,^^Y^	0.23±0.05^M^	0.12±0.02^1^^,^^M^^,^^Y^
**TUG (s)**	4.43±0.62	4.92±1.17^Y^	4.80±0.70	5.29±0.56^Y^
	LOADED			
**Peak Power (W)**	4147±698	2552±732^Y^	2442±415^M^	1503±165^M^^,^^Y^
**GRF (kN)**	2.11±0.27^1^	1.64±0.20^1^^,^^Y^	1.36±0.15^1^^,^^M^	1.24±0.17^1^^,^^M^
**V**_**off**_ **(m·s**^**-1**^**)**	2.45±0.22	1.75±0.35^1^^,^^Y^	2.03±0.19^M^	1.36±0.14^1^^,^^M^^,^^Y^
**Jump height (m)**	0.30±0.06	0.15±0.06^1^^,^^2^^,^^Y^	0.20±0.04^M^	0.09±0.02^1^^,^^2^^,^^M^^,^^Y^
**TUG (s)**	4.20±0.73	5.27±1.16^2^^,^^Y^	4.84±0.64	5.61±0.80^2^^,^^Y^

GRF: Ground reaction force; TUG: Timed up-and-go; V_off_: Take-off velocity;

^1^: Different from unloaded at *p*≤0.033;

^2^: Different form normal at *p*≤0.008;

^M^: Different from men at *p*<0.001;

^Y^: Different from young at *p*<0.001. Each group *n* = 10; data are mean±SD.

The peak ground reaction force (GRF) was higher in men than women (*p*<0.001). The sex × age interaction (*p* = 0.026) was reflected by a smaller GRF in old than young men (*p*<0.001) with no significant difference between old and young women. The GRF was lower in the unloaded than the normal and loaded conditions (*p*<0.001) with no significant difference between the normal and loaded condition (Table 2).

Men jumped higher than women, and young participants jumped higher than old participants (*p*<0.001). Irrespective of age or sex, the jump height was highest in the unloaded and lowest in the loaded conditions with the normal condition in between the two (*p*≤0.008).

The V_off_ was lower in the old than the young participants (*p*<0.001) and higher in men than women (*p*<0.001). The V_off_ was higher in the unloaded than the normal and loaded condition, irrespective of age and sex (*p*<0.001), but there was no significant difference in the V_off_ between the normal and loaded condition (Table 2).

There was no significant difference in TUG between men and women. Older participants performed the TUG slower than young participants (*p* = 0.007). The age × load interaction (*p* = 0.001) was reflected by a slowing of the TUG after loading in old (*p* = 0.004), but not in young participants, where, though not significant, the TUG of YM was even slightly faster in the loaded condition (Table 2).

### Determinants of timed up-and-go performance

**Fig 2:**
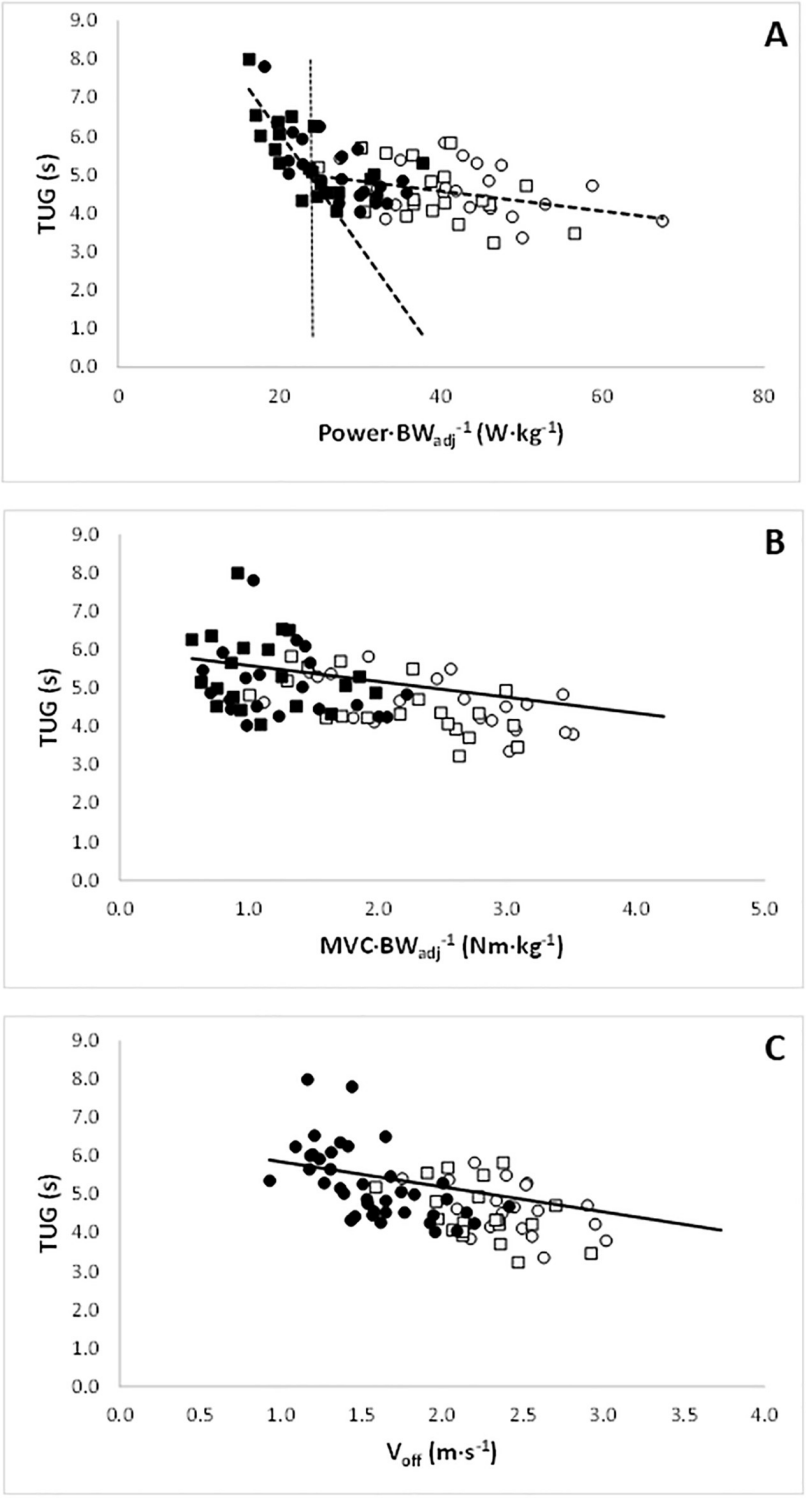
The Timed Up-and-Go (TUG) as a function of A) the peak power per adjusted body weight (BW_adj_, the weight including the harness and with or without the extra 15% body weight). The transition point occurs at 23.7 W·kg^-1^ (indicated by the vertical line), suggesting that if the power the muscles can generate decreases below the threshold of 23.7 W·kg^-1^ the TUG takes progressively longer; B) maximal isometric voluntary torque per body weight (MVC·BW_adj_) (*p* = 0.019) and C) take off velocity (V_off_) (*p*<0.01). ○: Young normal; ◻: Young loaded; ●: Old normal; ■: Old loaded.

In Fig 2A, the TUG is plotted as a function of the peak power per BW_adj_, (the weight including the harness and in the loaded condition it includes +15% body weight). To determine whether sub-regions existed with the TUG and body weight adjusted power data, a multi-variate adaptive regressive spline (MARS) [22] was fit to these data using the ‘earth’ package [23] in R. This approach automatically determines transition points, where the relationship between variables changes, provided in the derived hinge function of the form Max[0,|*c*–*x*|]. The resulting fit was described by:

$$5.00+(0.297\;\times \;\text{Max}[0,23.71-\text{Power}\cdot {\text{BW}}_{\text{adj}}{}^{-1})-(0.026\;\times \;\text{Max}[0,\text{Power}\cdot {\text{BW}}_{\text{adj}}{}^{-1}-23.71),$$

with *R*^2^ = 0.51 indicating 23.7 W·kg^−1^ as the transition point in these data (Fig 2A). This suggests that, in these data, if the power the muscles can generate decreases below the threshold of 23.7 W·kg^-1^ the TUG takes progressively longer.

As power is determined by force and velocity, we evaluated TUG as a function of MVC per BW_adj_ and V_off_, to identify the relationship between each of these components of power (i.e. force and velocity). The mixed effects model showed a significant effect of MVC per BW_adj_ (*p* = 0.019), but not age group (*p* = 0.440) for the prediction of TUG (Fig 2B). This was repeated when V_off_ was used as a predictor (*p* = 0.0096), with no effect of age group (*p* = 0.479; Fig 2C). Including both V_off_ and MVC per BW_adj_ in the model to predict TUG did not improve the model fit.

### Determinants of peak muscle power

**Fig 3:**
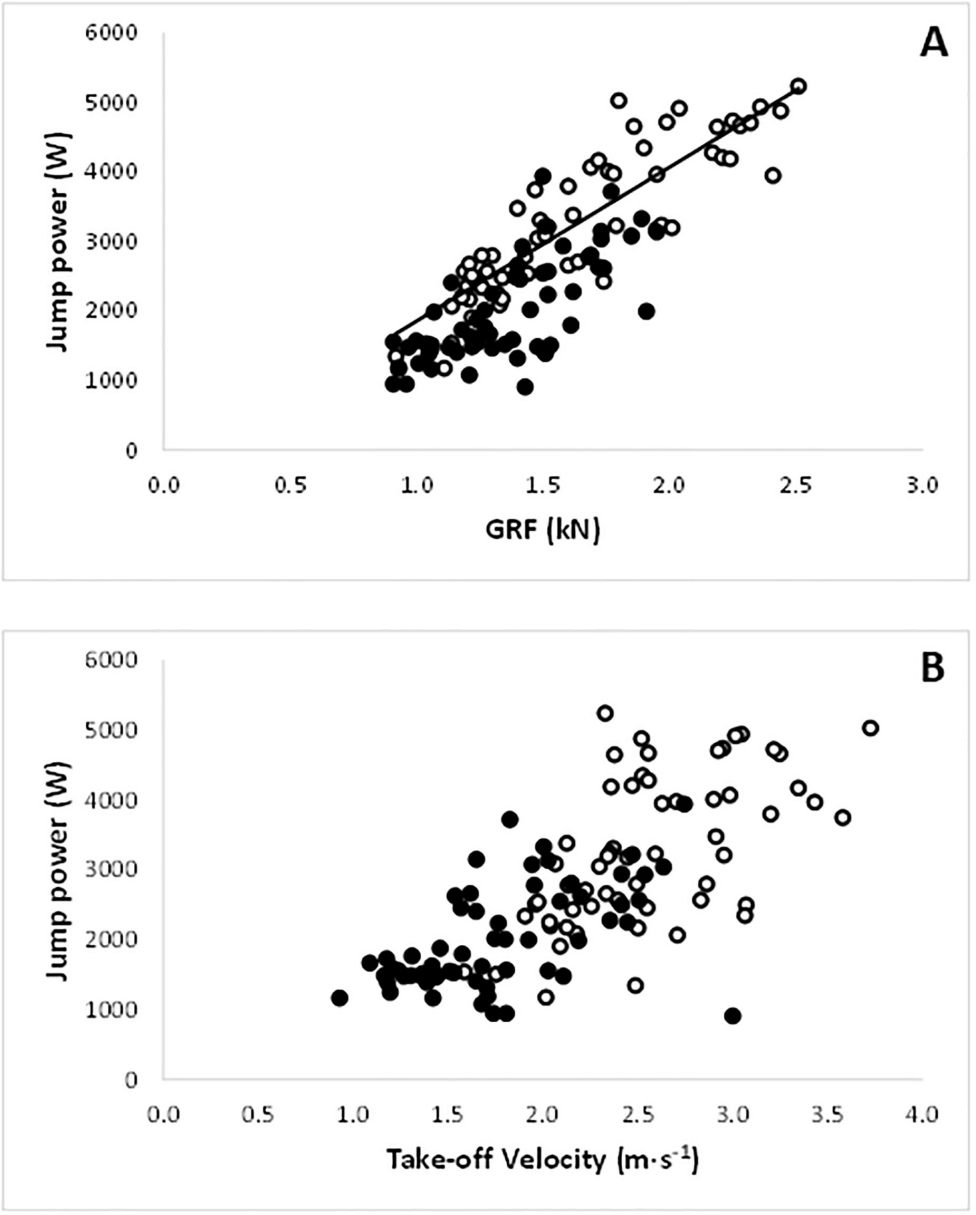
The peak power during a countermovement jump correlated with A) the peak ground reaction force (GRF) (correlation in young p<0.001; old: NS) and B) and take-off velocity (V_off_). ○: Young; ●: Old.

Power is the product of force and velocity. The mixed effect model showed there was a significant effect of age group (*p*<0.001) on the association between CMJ GRF and peak jump power. Fitting the model to the young age group showed a positive relationship between GRF and peak jump power (*p*<0.001), while for the old age group this relationship was not significant (*p* = 0.079) (Fig 3A).

When assessing CMJ peak power as a function of V_off_ (Fig 3B), the mixed effects model for the complete data set indicated age group (*p* = 0.0001), but not V_off_ was a significant predictor (*p* = 0.493).

**Fig 4:**
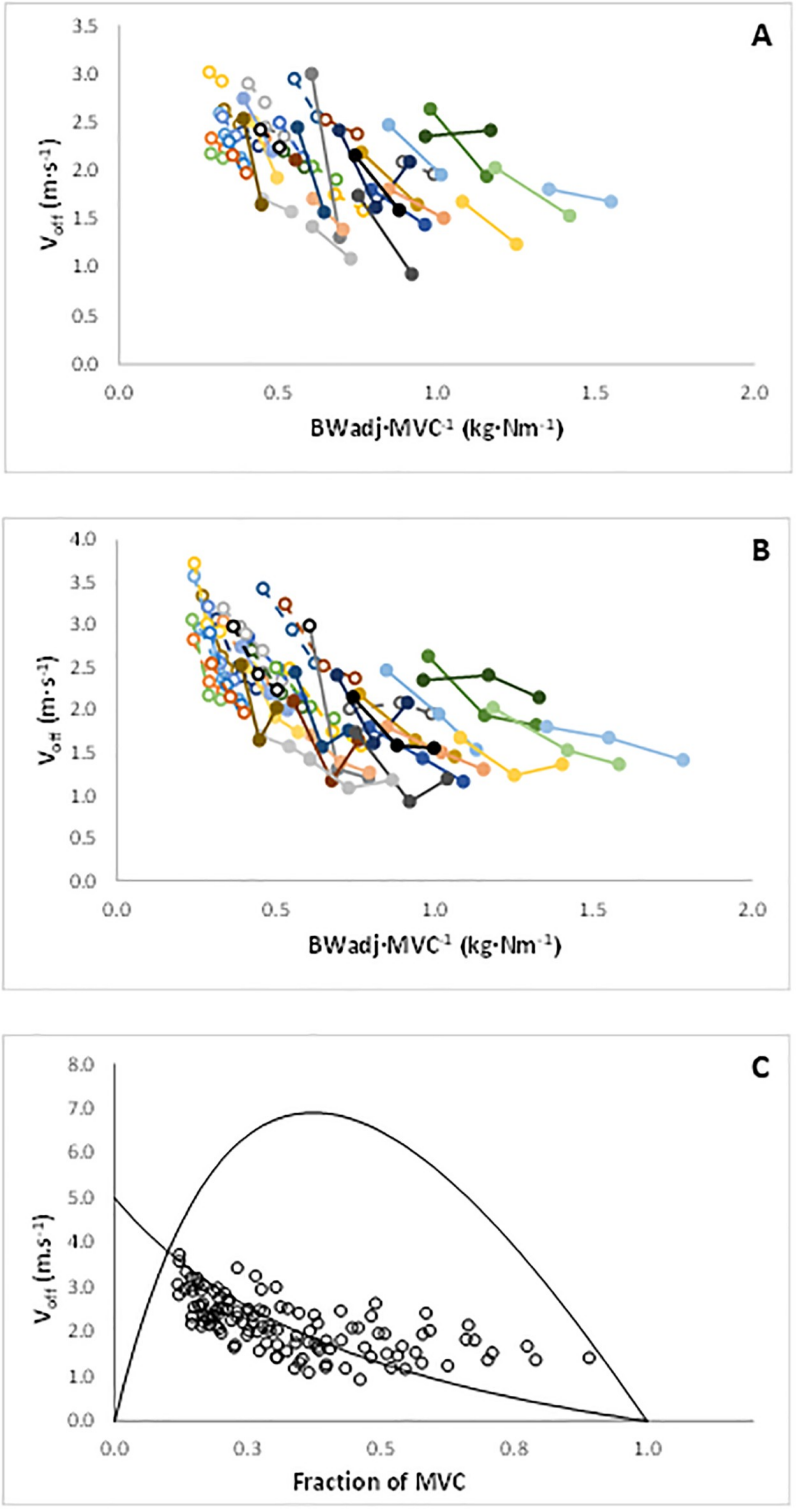
Take-off velocity (V_off_) as a function of adjusted body weight divided by maximal voluntary isometric torque (BW_adj_·MVC^-1^) in A) normal and loaded young (open symbols and dashed lines) and normal and unloaded old (closed symbols and solid lines) participants and in B) added the unloaded young and loaded old data. Black lines and open and closed symbols indicate the average for young and old, respectively. C) To fit the data with least squares analyses to the Hill equation, we divided BW_adj_·MVC^-1^ by 2 and found an R^2^ = 0.396.

According to the force-velocity relationship, the velocity of a muscle contraction is determined by the load on the muscle. Therefore, the lower V_off_ in old than young participants could be attributable to their lower MVC (Table 1). Although in some cases there was an unexpected increase in V_off_ with loading, as predicted by the force-velocity relationship we observed in the pooled data of all conditions a negative correlation between V_off_ and BW_adj_·MVC^-1^ (a reflection of P/Po in the force-velocity relationship) (R^2^ = 0.596; *p*<0.001) in both young (R^2^ = 0.309; *p*<0.001) and old (R^2^ = 0.086; *p* = 0.023) participants. In the control condition, the relationship appears different in young and old. Specifically, the V_off_ at a given BW_adj_·MVC^-1^ appears lower in old than young participants (Fig 4A). To explore whether this is related to slower contractile properties of young than old muscles, we loaded young people to make the BW_adj_·MVC^-1^ similar to that seen in old people and unloaded old people to make their BW_adj_·MVC^-1^ similar to that in young people. In Fig 4A it can be seen that the data points of the young loaded people shifted somewhat in the direction of the old people, and those of the unloaded old people overlapped those of the normal young people. In Fig 4B the data of the unloaded young and loaded old people are presented, and it can be seen that the data points form a continuum, with no clear gap between the data points from young and old people. A mixed effect model indicated that there was indeed no significant effect of age (*p* = 0.142), but there were effects of loading condition and BM_adj_·MVC^-1^ (both *p*<0.001). To fit the data with least squares analyses to the Hill equation, we normalised BW_adj_·MVC^-1^ and found an R^2^ = 0.396 (Fig 4C).

## Discussion

Our study confirmed that older people have a lower peak jumping power than younger people. It appeared that only if the peak power was below a critical threshold (23.7 W·kg^-1^) the TUG performance became progressively worse. The main observation of this study was that the lower power generating capacity of older than young people was primarily due to a loss of force generating capacity, rather than a slowing of the contractile properties of the muscle, as reflected by the similar relationship between jump take off velocity (V_off_) and body weight (adjusted) to MVC ratio in young-adults and older participants (Fig 4C).

### Age-related differences in TUG

It has been reported that loss of muscle function is a more important determinant of physical functioning than muscle mass [9]. In fact, in healthy older people no significant correlation between the performance of TUG and the 6-minute walking test (6MWT) with muscle mass was found, while there was a significant correlation with peak muscle power [1]. In the latter study, however, those correlations existed only in older, but not in young-adult participants, which may indicate that only if the power generating capacity drops below a threshold TUG and 6MWT performance are limited. Here we tested this by assessing the impact of loading—reducing the peak power per body weight + load (BW_adj_)—on TUG. The overlap of the data TUG *vs*. peak power per BW_adj_ of the loaded young-adults with those of the normal and unloaded older people indeed suggests that only if peak power per body mass drops below a critical threshold (here 23.7 W·kg^-1^) TUG performance is impaired. This transition point is somewhat sensitive on the type of analysis and needs to be confirmed in a larger study population. Nevertheless. this cut-off in the CMJ may serve as an early indicator of developing of sarcopenia and suggests that this easy test can help to identify people that may benefit from an early intervention to prevent or delay the development of sarcopenia [8].

The lower TUG performance in the old is primarily attributable to a loss of power generating capacity, as we did not see a significant difference in body weight between the old and young-adult participants. Such an age-related reduction in peak jumping power has been seen before in men and women [1,7], and even in master athletes [24,25]. This lower power can be the result of a lower maximal force generating capacity and/or slower contractile properties of muscles from older rather than young-adult people [16].

### Age-related differences in peak power

Similar to previous observations [1], the peak power for a given ground reaction force (GRF) was higher in young-adult than old participants (Fig 3A). As power is the product of force and velocity, the greater power for a given GRF in the young is attributable to their higher V_off_ (Fig 3B). At first glance, this suggests that both a lower force generating capacity and slower contractile properties contribute to the lower power generating capacity in the old. However, the GRF did not differ significantly between normal young-adult and old participants, even though the MVC was almost 50% lower in the old, and as seen before correlates with body mass [1] rather than the force generating capacity of the muscle. The lower V_off_ of the old, having a similar body mass as the young-adults, may therefore be a consequence of the muscle working at a higher proportion of the maximal force generating capacity and hence a slower part of the force-velocity relationship during a CMJ [16]. However, as the V_off_ *vs*. BW_adj_/MVC ratio did not show any overlap between younger and older people and the data points were on two different and parallel regression lines [1], it was concluded that the lower peak jump power in old individuals was at least partly attributable to a slowing of the muscle contractile properties.

While the argument sounds rather convincing and corresponds with other studies showing a reduced optimal velocity during leg press [15] and suggested mechanism of slowing of muscle, such as slowing of type I fibres and a slow-to-fast shift in fibre type composition [26–28], it remains to be seen to what extent the apparent slowing in old age is really attributable to slower contractile properties. In fact, there are studies showing no slowing of muscle fibre contractile properties [29], nor an age-related fast-slow transition in fibre type composition [30]. One way to test whether the contractile properties of the muscle are slower in old age is to increase the load on the muscle during a CMJ in the young and decrease the load in older people, so both act on a similar portion of the force-velocity relationship.

Such loading and unloading CMJ studies have been performed before in young adults [19,31] and as expected it was found also here that unloading resulted in an increased jump height. While in a previous study in young men unloading was accompanied with an increased peak power [19], in our study there was no significant effect of load on power. The discrepancy may be attributable to the knee extensor torque during an MVC, where it has been shown that the magnitude of the differences in peak power between loading conditions decreases with decreasing maximal knee extensor torque [19], where the torque of our participants was in the lower, and even below the, range of their participants. Whatever the explanation of this discrepancy, the increased [19] or maintained peak power during unloading must be accompanied by an increase in V_off_, as has indeed been seen by others [31] and in the current study. This increase in V_off_ with unloading is explicable by the unloaded muscle working on a faster portion of the force-velocity relationship rather than a loading-induced change in muscle contractile properties.

While in Fig 4A the V_off_ for a given BW_adj_·MVC^-1^ appears higher for young than old participants, similar to that seen previously [1], loading of the young and unloading of the old led to a significant overlap of the data points (Fig 4B). In Fig 4C we added the young loaded and old unloaded data, creating a continuous graph without any non-overlapping clusters, similar to the impact of unloading on V_off_ discussed in the previous paragraph. This suggests, in contrast to the conclusion that older people have slower contractile properties [1], that the lower V_off_ in a CMJ in older people is attributable to their weaker muscles working at a slower portion of the force-velocity relationship, rather than slower contractile properties.

To explore this further we fitted the data to the Hill equation and found that it explained 40% of the variation in the data. It is noticeable that the deviation from the force-velocity relationship occurs particularly at the higher loads, where the V_off_ is higher than expected. This was somewhat unexpected, as it has been seen that during higher loads the depth of the CMJ is decreased [19] and consequently the acceleration upward is over a shorter distance [31] and consequently one would expect a lower, rather than a higher, V_off_. One possible explanation for the higher than expected V_off_ at high loads is that throughout the movement the muscle will work at a slower part of the force-velocity relationship and hence, all else staying the same, this increases the time for acceleration. It is thus possible that the increased time for acceleration more than offsets the lesser distance of acceleration.

### Limitations

In theory, a higher tendon stiffness in old age could obscure slowing contractile properties of muscles, but this is unlikely to be the case as it has been observed that tendon stiffness decreases with age [32]. In addition, we did not consider the storage and use of elastic energy in the CMJ. However, this appears to play a minor role in the jump performance and it is rather potentiation of cross-bridges that enhances performance in a CMJ above that of a static jump [33]. In addition, the difference in mass-specific peak jumping power between marmosets and humans was explicable by differences in fibre type composition and the force-velocity relationship of the fibres, suggesting that the CMJ gives a fair representation of muscle contractile properties [34]. While reduced balance in old age [32] and joint pain may limit muscle activation and jump performance, it is unlikely that this caused major issues in our population, given the considerable overlap in loaded young and unloaded old data. Another issue, as alluded to above, is the depth of a CMJ that not only affects the duration of acceleration, but also the leverage of the muscles used for jumping that all may have an impact on jump performance. A potential limitation of the study is therefore a lack of standardisation of the depth of the countermovement. In addition, the YM, though not significant, performed the TUG better in the loaded condition and in some participants the V_off_ was increased rather than decreased with increased load. This may be attributable to problems with coordination during the jump, or working just below the peak of the power-velocity curve during normal loading. Nevertheless, ~40% of the data was explicable by the force-velocity relationship. Although ideally the force-velocity should be calculated for each individual separately, this was not possible with just three datapoints with a narrow spread. However, the overlap of male and female and young and old data suggest that the force-velocity relationship was not different between groups. In addition, it was particularly the loaded condition of the young participants that deviated from the Hill curve, a condition that had no bearing on the argument developed here that normal young and old people work on different parts (slow and fast, respectively) of the force-velocity curve.

### Conclusion

The lower peak jumping power, indicative of leg muscle power generating capacity, is lower in old than young people and will have a negative impact on the functioning of daily life activities when it drops below a critical threshold. The lower take-off velocity of a jump in the old than the young was explicable by their lower muscle strength rather than slower contractile properties, forcing their muscles to work on a slower part of the force-velocity relationship. These data indicate that muscle strengthening exercise or interventions are only effective to improve daily life if power is below a critical threshold.
